# Supplementary material for: Follicular Fluid Proteomic Analysis of Women Undergoing Assisted Reproduction Suggests That Apolipoprotein A1 Is a Potential Fertility Marker
Source: Int J Mol Sci. 2023 Dec 29;25(1):486. doi: 10.3390/ijms25010486 (PMC10778837; doi:10.3390/ijms25010486)
Supplement: Supplementary file 1 [file ijms-25-00486-s001.zip › ijms-2766712-supplementary.pdf]

Supplementary Table S1. Abbreviations of the follicular fluid proteins in the PPI analyses.

**Abbreviation**	**Name of the protein**
A1BG	Alpha-1B-glycoprotein
A2M	Alpha-2-macroglobulin
AFM	Afamin
AGT	Angiotensin
AHSG	Alpha-2-HS-glycoprotein chain A
ALB	Serum albumin
AMBP	Inter-alpha-trypsin inhibitor
APOA1	Apolipoprotein A-I
APOA2	Apolipoprotein A-II
APOA4	Apolipoprotein A-IV
APOC3	Apolipoprotein C-III
APOH	Beta-2-glycoprotein 1
APOL1	Apolipoprotein L1
C3	Complement C3c alpha chain fragment 1
C4B	Complement C4-B alpha chain
C5	Complement C5 alpha chain
C8B	Complement component C8 beta chain
C9	Complement component C9a
CFB	Complement factor B Ba fragment
CFH	Complement factor H
CLU	Clusterin alpha chain
CP	Ceruloplasmin
CPN2	Carboxypeptidase N subunit 2
F2	Activation peptide fragment 1
FGA	Fibrinogen alpha chain
FGB	Fibrinogen beta chain
FGG	Fibrinogen gamma chain
FN1	Fibronectin
GC	Vitamin D-binding protein
GSN	Gelsolin
HBA1	Hemoglobin subunit alpha 1
HBB	Hemoglobin subunit beta
HP	Haptoglobin alpha chain
HPX	Hemopexin
HRG	Histidine-rich glycoprotein
HSPG2	Basement membrane-specific heparan sulfate proteoglycan core protein
IGHV3OR16-9	Immunoglobulin heavy variable 3/OR16-9
IGKV2D-28	Immunoglobulin kappa variable 2-28
ITIH1	Inter-alpha-trypsin inhibitor heavy chain H1
ITIH2	Inter-alpha-trypsin inhibitor heavy chain H2
ITIH4	35 kDa inter-alpha-trypsin inhibitor heavy chain H4
KNG1	Low molecular weight growth-promoting factor
LBP	Lipopolysaccharide-binding protein
LRG1	Leucine rich alpha-2-glycoprotein 1
ORM1	Alpha-1-acid glycoprotein 1
PGLYRP2	N-acetylmuramoyl-L-alanine amidase
PLG	Plasmin heavy chain A, short form

PON1	Serum paraoxonase/arylesterase 1
SERPINA1	Short peptide from AAT
SERPINA3	Alpha-1-antichymotrypsin His-Pro-less
SERPINA6	Corticosteroid-binding globulin
SERPINA7	Thyroxine-binding globulin
SERPINC1	Antithrombin-III
SERPIND1	Heparin cofactor 2
SERPINF1	Pigment epithelium-derived factor
SERPINF2	Alpha-2-antiplasmin
SERPING1	Plasma protease C1 inhibitor
SHBG	Sex hormone-binding globulin
TF	Serotransferrin
TTR	Transthyretin
VTN	Vitronectin V10 subunit
WHAMM	WASP homolog-associated protein with actin, membranes and microtubules
